# Routine perioperative blood tests predict survival of resectable lung cancer

**DOI:** 10.1038/s41598-023-44308-y

**Published:** 2023-10-10

**Authors:** Daniele Morelli, Anna Cantarutti, Camilla Valsecchi, Federica Sabia, Luigi Rolli, Giovanni Leuzzi, Giorgio Bogani, Ugo Pastorino

**Affiliations:** 1 Department of Pathology and Laboratory Medicine, Fondazione IRCCS Istituto Nazionale dei Tumori, Milan, Italy; 2 Division of Biostatistics, Epidemiology and Public Health, Department of Statistics and Quantitative Methods, University of Milano-Bicocca, Milan, Italy; 3 Division of Thoracic Surgery, Fondazione IRCCS Istituto Nazionale dei Tumori, Via Venezian 1, 20133 Milan, Italy; 4 Department of Gynecologic Oncology, Fondazione IRCCS Istituto Nazionale dei Tumori, Milan, Italy

**Keywords:** Lung cancer, Risk factors

## Abstract

There is growing evidence that inflammatory, immunologic, and metabolic status is associated with cancer patients survival. Here, we built a simple algorithm to predict lung cancer outcome. Perioperative routine blood tests (RBT) of a cohort of patients with resectable primary lung cancer (LC) were analysed. Inflammatory, immunologic, and metabolic profiles were used to create a single algorithm (RBT index) predicting LC survival. A concurrent cohort of patients with resectable lung metastases (LM) was used to validate the RBT index. Charts of 2088 consecutive LC and 1129 LM patients undergoing lung resection were evaluated. Among RBT parameters, C-reactive protein (CRP), lymphocytes, neutrophils, hemoglobin, albumin and glycemia independently correlated with survival, and were used to build the RBT index. Patients with a high RBT index had a higher 5-year mortality than low RBT patients (adjusted HR 1.93, 95% CI 1.62–2.31). High RBT patients also showed a fourfold higher risk of 30-day postoperative mortality (2.3% vs. 0.5%, p 0.0019). The LM analysis validated the results of the LC cohort. We developed a simple and easily available multifunctional tool predicting short-term and long-term survival of curatively resected LC and LM. Prospective external validation of RBT index is warranted.

## Introduction

Lung cancer (LC) is one of the most common malignancies in developed countries. In 2023, 238,000 LC cases and 127,000 cancer-related deaths are projected to occur in the United States^1^. Despite the introduction of innovative treatment modalities (e.g., immunotherapy, targeted therapy), the lethality (death on incidence ratio) of LC remains very high, with an estimated five-year overall survival ranging between 15 and 19%^2^.

To date, prognosis in lung cancer relies only on tumor-related factors, summarized by the TNM staging system^3^. Accumulating evidence highlighted that several factors might impact patient outcomes. Over the last decade, several constitutional, hormonal, and humoral factors have been associated with patients' prognoses. Many studies suggested that age, sex, smoking history, performance status, and nutritional habits influence tumor behavior. Similarly, the association between inflammatory status and cancer outcome is under investigation^4–6^. Some inflammatory markers, such as C-reactive protein (CRP), neutrophils, neutrophils/lymphocyte ratio (NLR), and lymphocyte/monocyte ratio (LMR), have been shown to play an important role in the prognosis of patients with cancer^7–9.^

Metabolic status is another important index for cancer management^10^. Jafri et al., developed an advanced lung cancer inflammation index (ALI) that combined body mass index (BMI) and albumin with NLR^10^. Several studies have corroborated these findings, where low ALI scores are associated with a poor prognosis in various solid tumors (including lung cancer)^11–14^. Valero et al., recently proposed a novel index (Host-Index) that included parameters such as neutrophils, monocytes, hemoglobin, albumin, and lymphocytes. This H-index showed a good prognostic capacity in a cohort of patients with oral cavity cancer and neck squamous cell carcinomas^15,16^

Despite the increasing evidence of the effect of the inflammatory and metabolic index on the prognosis of cancer, there is no robust evidence supporting the adoption of a composite prognostic index that can be used in clinical practice.

Hence, we designed the present study to combine different predictors of survival in resectable lung cancer. Through the evaluation of pre-and postoperative routine blood tests (RBT) depicting inflammatory, immune and metabolic profiles we developed a multifunctional prognostic index, using a large dataset of patients undergoing surgical resection of primary lung cancer (LC). The value of RBT was then validated in a second data set of patients undergoing surgical resection lung metastases (LM) from other primary tumors.

## Methods

This is a single-center retrospective study. The Institutional Review Board (IRB) approval was obtained. All patients included signed informed consent for research purposes. Inclusion criteria were: (1) age ≥ 18 years; (2) signed informed consent; (3) the execution of thoracic surgical resection; (4) available details about RBT; and (5) diagnosis of invasive lung tumors (either primary or secondary (i.e., metastasizes from other solid tumors)). Exclusion criteria were: (1) absence of tumor tissue on lung specimen; and (2) consent withdrawal.

The main outcome measure of this study was to identify a prognostic score able to predict 5-year survival in patients undergoing thoracic resection for cancer purposes.

### Study population

The cohort consisted of all patients with primary LC or lung metastasis (LM) who underwent complete anatomical resection or lung metastasectomy with curative intent at the Thoracic Surgery Division of the Fondazione IRCCS Istituto Nazionale dei Tumori di Milano (Milan, Italy) from January 01, 2003, to December 31, 2017^17^. Only the first procedure has been evaluated, when patients had multiple surgical procedures. Patients were staged according to the 8th edition of the International Association for the Study of Lung Cancer (IASLC) staging system^18^.

### Data collection and follow-up

**Figure 1:**
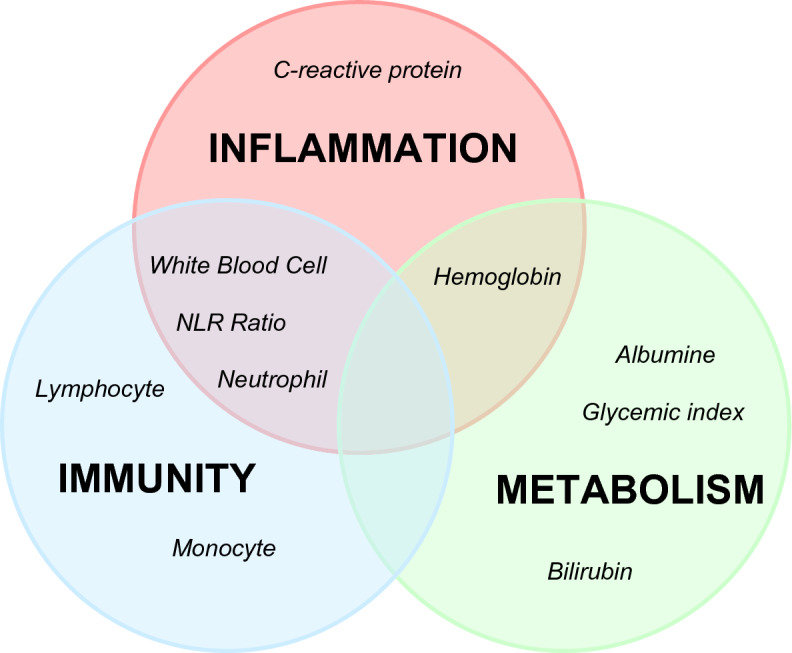
Routine blood test index profile.

Available data at baseline (before the surgery date) were gender, age, and pathological TNM staging. Moreover, blood samples were collected at baseline for examination of (a) inflammatory profile based on plasma level of C-Reactive Protein (CRP, mg/dL) and neutrophil–lymphocyte ratio (NLR,), (b) immunological profile, based on monocytes (10^3^/μL), neutrophils (10^3^/μL), lymphocytes (10^3^/μL), and the number of white blood cells (WBCs/µL), and (c) metabolic profile, based on hemoglobin (g/dL), glycemic index (mg/dL), and bilirubin (mg/dL) (Fig. 1). We considered only blood tests performed within 7 days before surgery (− 7d). Only albumin was routinely tested three days after surgery (g/dL). CRP, glucose, bilirubin and albumin were quantified using a Roche automated clinical chemistry analyzer (Roche Diagnostics, Mannheim, Germany); data of blood cell population counts were collected with an automated blood cell analyzer (ADVIA 2120i; Siemens Healthcare Diagnostics, Marburg, Germany). Each individual accumulated person-years of follow-up from baseline until censoring, i.e., the outcome onset (all causes of mortality) or the end of the study. The vital status and date of death were obtained through the Istituto Nazionale di Statistica (ISTAT, SIATEL 2.0 platform). Participants accumulated person-years of follow-up from the date of surgery until death or the date of the last follow-up as of December 2022.

### Statistical analysis

Descriptive statistics of socio-demographic and clinical characteristics of primary LC and LM were reported as frequencies (proportions) for categorical variables and median and Interquartile range (IQR) for continuous variables. The chi-square test and the Wilcoxon-Mann–Whitney test were used appropriately. All analyses were performed using the Statistical Analysis System Software (version 9_4; SAS Institute, Cary, NC). Statistical significance was set at the 0.05 level. All p values were two-sided.

### Score development

To select blood parameters of inflammatory, immunological, and metabolic profiles able to predict five-year mortality (i.e., the main outcome of interest), we proceeded as follows.

A training set containing patients with primary LC was used to develop the score, which was then validated in patients with lung metastases (validation set).

First, the outcome-oriented method proposed by Contal and O’Quigley based on the log-rank test statistics was used to select the single optimal cut-point for each blood parameter (Figure S1)^19–21^. For each profile of interest, with the aim of selecting variables independently able to predict survival, the stepwise selection method was applied, via Cox’s proportional hazard regression model in the training set.

The relationship between the selected parameters and the time to death was investigated by fitting parametric survival models based on the Weibull distribution. The coefficients estimated from the model were used for assigning a weight to each selected covariate for each profile of interest. In particular, a weight was assigned to each coefficient by multiplying it by 10 and rounding it to the nearest whole number^22,23^. The weights thus obtained were then summed to produce the total aggregate score for each profile of interest. Finally, each profile was dichotomized using the outcome-oriented method described above, categorizing patients into low and high profiles.

Second, the prognostic value of age, gender, and stage of the tumor, as well as of each profile of interest in predicting 5-year mortality, was investigated by fitting Cox’s proportional hazard regression models. The inflammatory, immunologic, and metabolic profiles were separately and overall investigated. The area under the receiver operating characteristics curve (AUC), a summary measure of discrimination, was used as a primary performance metric of the considered models. We have also reported other metrics of improvement in fit, including the likelihood ratio test (for nested regression models). Model adjustments were made for age (> 70 years), gender, and TNM stage (I, II, or III-IV).

Finally, we developed the routine blood test (RBT) index as the sum of the inflammatory, immunologic, and metabolic profiles, and then we categorized it by assessing increasing values of low, intermediate, and high to categories of the aggregate score of 0–11, 12–17, and ≥ 18, respectively (Supplementary Sect. 1). The predictive performance of the RBT index was initially evaluated in the training set by constructing the receiver operating characteristic (ROC) curve and calculating the corresponding underlying area (area under the ROC curve (AUC)). The prognostic value of the RBT index was then investigated. Finally, the cumulative risk of overall survival at five years, overall and stratified by the TNM stage, was estimated using the Kaplan–Meier method, and time-to-event comparisons were made using the log-rank test for each contrast of interest^24^.

### Score validation

To assess the reproducibility and generalizability of the results, the RBT index was externally validated by applying it to patients with LM using the same inclusion/exclusion criteria as the primary LC patients. The predictive performance was assessed through discrimination and calibration. The discriminatory power was assessed by calculating the ROC curves and the corresponding AUC. Calibration plots displayed observed versus predicted overall survival at five years probabilities.

### Ethics approval and consent to participate

The blood sample for this study were collected during standard surgical procedures at Fondazione IRCCS Istituto Nazionale dei Tumori di Milano. Institutional approval from the Independent Ethics Committee of Fondazione IRCCS Istituto Nazionale Tumori was obtained for the conduct of this study. Patients agreed to the use of their own samples with informed consent. All data were analyzed anonymously, and all experiments complied with the 1975 Declaration of Helsinki.

## Results

### Study population

**Figure 2:**
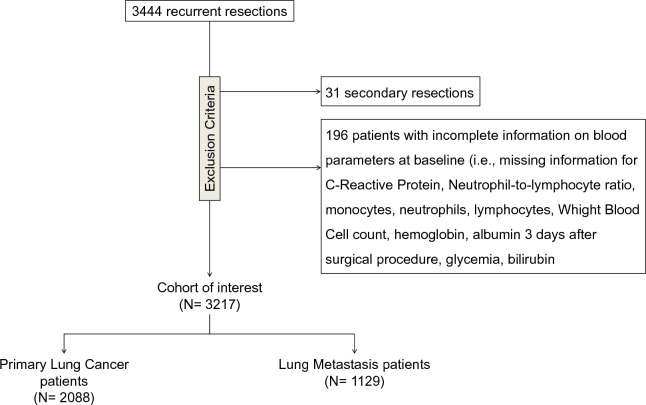
Flow-chart of the study.

Of 3444 consecutive lung resections, we excluded patients who had multiple surgical procedures (N = 31) and those without complete information on blood samples at baseline (N = 196). The final cohort consisted of 3217 patients who underwent their first resection during the study period. Out of 3217 patients, 2088 (65%) and 1129 (35%) had a primary LC and LM, respectively. Median follow-up of alive patients was 6 years in both LC and LM cohorts. Figure 2 shows the flow of patients through the study design.

The socio-demographic, clinical, inflammatory, immunological, and metabolic profile characteristics of LC and LM patients are shown in Table 1. The primary tumor site of LM cohort was reported in Table S1.

**Table 1:** Socio-demographic, clinical, and inflammatory, immunological and metabolic profiles description among primary lung cancer and metastasis.

	Primary lung cancer		Lung metastases		p-value
	(N = 2088)		(N = 1129)		
	N	(%)	N	(%)	
Age at surgery in yr.—median (IQR)	67.0	(13.2)	58.3	(20.8)	< .0001
Gender					
Female	706	(33.8)	466	(41.3)	< .0001
Male	1382	(66.2)	663	(68.7)	
Stage					
I	1002	(48.0)	–		
II	449	(21.5)	–		
III–IV	637	(30.5)	–		
Inflammatory markers					
CRP ≥ 3 mg/dL	1112	(53.3)	467	(41.4)	< .0001
NLR ≥ 2.9	895	(42.9)	489	(43.3)	0.8062
Immunology markers					
Mono. ≥ 0.32 × 10^3^/µL	1381	(66.1)	565	(50)	< .0001
Neu. ≥ 5 × 10^3^/µL	836	(40)	252	(22.3)	< .0001
Lymph. < 1.8 × 10^3^/µL	1127	(53.9)	794	(70.3)	< .0001
WBC ≥ 8130 /µL	671	(32.1)	170	(15.1)	< .0001
Metabolic markers					
Hb. < 13.5 g/dL	813	(38.9)	388	(34.4)	0.0105
Alb. < 3.14 g/dL	784	(37.6)	188	(16.7)	< .0001
Gly. ≥ 106 mg/dL	741	(35.5)	332	(29.4)	0.0005
Bil. < 0.5 mg/dL	1027	(49.2)	487	(43.1)	0.0010

Overall cohort, N = 3217.

### Routine blood test (RBT) index

We developed and (externally) validated the RBT index through the training and validation sets composed of primary LC and LM patients, respectively. Blood sample parameters that mainly contributed to the total aggregate profiles were (1) CRP (cut-off: ≥ 3 mg/dL; associated scores: 5) and NLR (≥ 2.9; 3) for the inflammatory profile; (2) monocytes (≥ 0.32 × 10^3^/µL; 4), lymphocytes (< 1.8 × 10^3^/µL; 4), and neutrophils (≥ 5 × 10^3^/µL; 2) in the immunologic profile; and (3) hemoglobin (< 13.5 g/dL; 5), albumin at three days post-surgery (< 3.14 g/dl; 5), and glycemic index (≥ 106 mg/dL; 3) in the metabolic profile. Albumin at three days after surgery showed a significant predictive value for overall survival at five years (Supplementary Sect. 2).

### Short term outcomes

Looking at short-term outcomes, we observed that in the LC cohort, RBT index predicted 30- and 90-day mortality. 90-day mortality in the low-, intermediate-, high-RBT score for LC patients was 1.2%, 1.4%, 5.2%, respectively (p < 0.001). Similarly, 90-day mortality in the low-, intermediate-, high-RBT score for LM patients was 0.40%, 0.40%, 2.2%, respectively (p = 0.02). Table S2 shows details regarding 30- and 90-day mortality.

### Long term outcomes

The correlation of socio-demographic, clinical, and inflammatory, immunological and metabolic markers with 5-year survival is shown in Table S3 (LC) and S4 (LM). In LC cohort 836 (40%) patients died within five years, compared to 529 (47%) patients in LM cohort. LC patients who died within 5 years were older, more likely to be male, with advanced tumor stage, higher inflammatory, immunologic, and metabolic markers described by several blood parameters in comparison to alive patients (Table S3). In LM cohort, only inflammatory (CRP, NLR) and some metabolic markers (Hb, Albumin, Glycemia) were associated with outcome (Table S4).

Patients were classified as having lower or higher inflammatory, immunologic, and metabolic profiles if each total aggregate score was equal to or greater than 4. Cox's proportional hazard model was used to analyze the prognostic value on 5-year overall survival of gender, age, stage of the tumor, and each profile, separately and altogether, through nested models (Table 2).

**Table 2:** The prognostic value of age, gender, stage of the tumour, and inflammatory, immunological, and metabolic profiles in predicting 5-year mortality.

	Mod 1	Mod 2	Mod 3	Mod 4	Mod 5	Mod 6
	HR (95% CI)	HR (95% CI)	HR (95% CI)	HR (95% CI)	HR (95% CI)	HR (95% CI)
Gender						
Female	1.00 (Reference)	1.00 (Reference)	1.00 (Reference)	1.00 (Reference)	1.00 (Reference)	1.00 (Reference)
Male	1.64 (1.40–1.92)	1.58 (1.35–1.85)	1.49 (1.27–1.75)	1.53 (1.31–1.80)	1.65 (1.41–1.93)	1.52 (1.29–1.79)
Age						
< 70 yr	1.00 (Reference)	1.00 (Reference)	1.00 (Reference)	1.00 (Reference)	1.00 (Reference)	1.00 (Reference)
≥ 70 yr	1.51 (1.32–1.73)	1.70 (1.48–1.95)	1.67 (1.46–1.92)	1.67 (1.46–1.92)	1.62 (1.41–1.86)	1.59 (1.38–1.83)
Stage						
I		1.00 (Reference)	1.00 (Reference)	1.00 (Reference)	1.00 (Reference)	1.00 (Reference)
II		2.16 (1.78–2.61)	2.00 (1.65–2.43)	2.14 (1.76–2.59)	2.00 (1.65–2.43)	1.88 (1.55–2.29)
III-IV		4.08 (3.47–4.8)	3.98 (3.39–4.69)	4.04 (3.44–4.76)	3.77 (3.19–4.44)	3.70 (3.13–4.36)
Inflammatory score¥						
Low			1.00 (Reference)			1.00 (Reference)
High			1.55 (1.35–1.79)			1.47 (1.27–1.70)
Immune score¦						
Low				1.00 (Reference)		1.00 (Reference)
High				1.41 (1.06–1.86)		1.31 (0.99–1.73)
Metabolic score§						
Low					1.00 (Reference)	1.00 (Reference)
High					1.49 (1.29–1.73)	1.41 (1.21–1.64)
*AUC*	*0.61*	*0.74*	*0.75*	*0.75*	*0.75*	*0.76*
*-2 Log L*	*12,254.240*	*11,949.321*	*11,911.992*	*11,943.009*	*11,920.599*	*11,877.591*

Primary lung cancer cohort (N = 2088).

^¥^Inflammatory profile included CRP (cut-off < 3) with a score of 5, and NLR (< 2.9) with a score of 3.

^¦^Immune profile included monocytes (< 0.32) with a score of 4, lymphocytes (≥ 1.8) with a score of 4, and neutrophils (< 5) with a score of 2.

^§^Metabolic profile included haemoglobin (≥ 13.5) with a score of 5, albumin at three days post surgery (≥ 3.14) with a score of 5, and glycemic index (< 106) with a score of 3.

**Figure 3:**
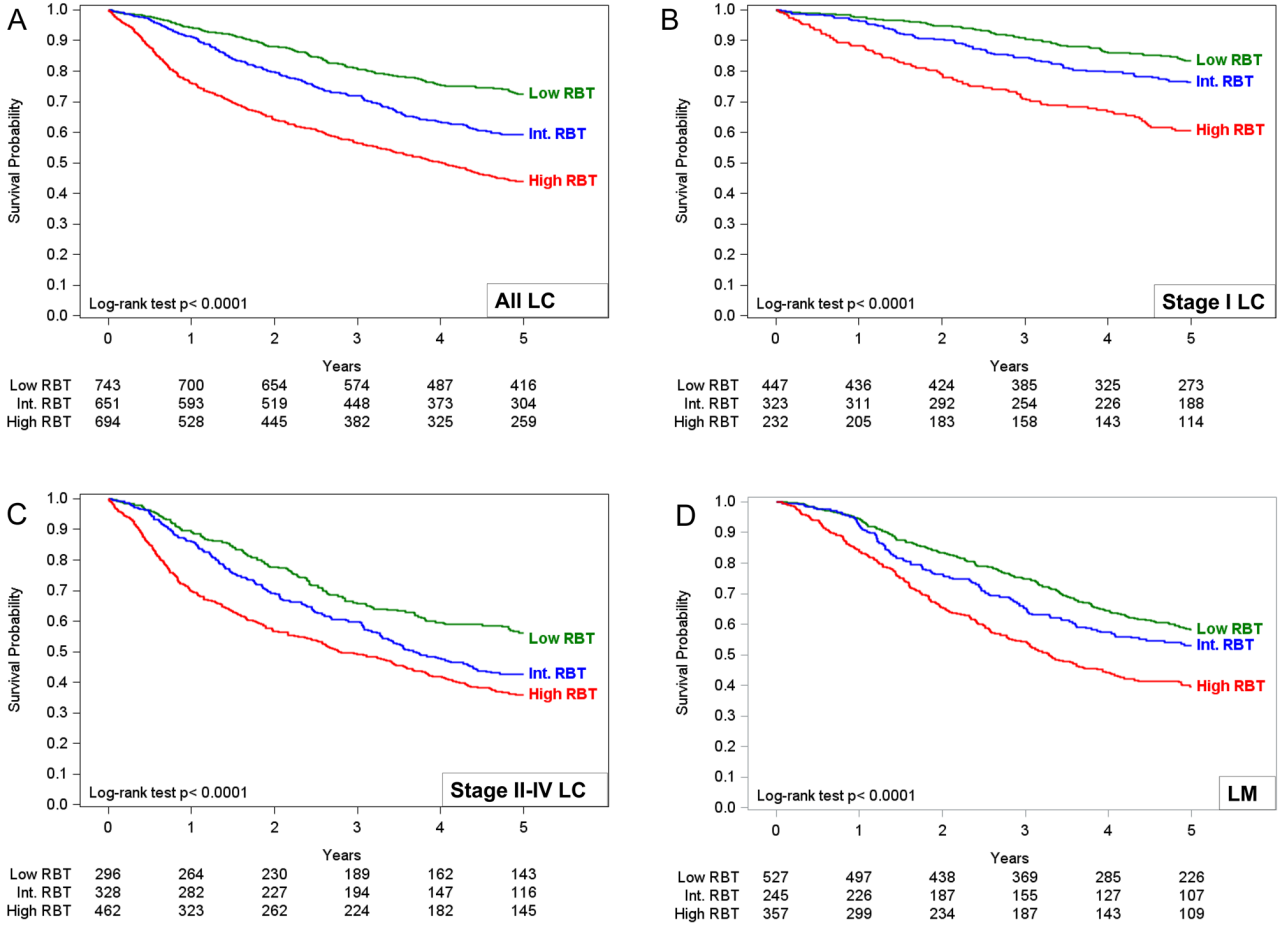
Overall survival curves according to the RBT index cut-offs; (**A**) LC cohort; (**B**) LC cohort stratified for stage I disease; (**C**) LC cohort stratified for stage II–IV disease; (**D**) LM cohort.

Results showed the independence of the variables. As expected, the full model showed a significant effect of gender (HR 0.66, 95% CI 0.56–0.77), age (1.59, 1.38–1.83), tumor stage (compared to stage I, stage II: 1.88, 1.55–2.29; stage III–IV: 3.70, 3.13–4.36), inflammatory profile (1.47, 1.27–1.70), immunologic profile (1.31, 0.99–1.73), and metabolic profile (1.41, 1.21–1.64) (difference in -2 Log-L models 3 and 6 = 11,877.264–11,911.992 = 34.7 degrees of freedom, p < 0.0001, AUC = 76%). Subsequently, we added all the profiles described above to develop the RBT index and categorized patients based on having low (score ≤ 11), intermediate (score between 12 and 17), and high (score ≥ 18) RBT index. Compared with patients with a low RBT index, patients with a high RBT level had a higher risk of a worse 5-year outcome (adjusted HR 1.93, 95% CI 1.62–2.31), as well as patients with an intermediate level (1.34, 1.11–1.61) (Table 3). The results were consistent with stratified analysis by the tumor stage and among patients with lung cancer metastasis (Fig. 3, and S2). Multivariate Cox model stratified for the LM cohort showed a worse 5-years survival outcome in patients with high RBT index compared to patients with low RBT index (adjusted HR 1.88, 95% CI 1.54–2.28) (Table S5). Interestingly, the RBT index provided a similar level of efficacy in predicting outcomes when preoperative albumin levels were used instead of the postoperative ones in those patients where both values had been measured (data not shown).

**Table 3:** Multivariate Cox proportional hazards model to assessed the prognostic value of age, sex, tumor stage, and RBT score in predicting 5-year mortality in the primary lung cancer cohort (N = 2088).

	HR (95% CI)
Gender	
Female	1.00 (Reference)
Male	1.50 (1.28–1.76)
Age	
≤ 70 yr	1.00 (Reference)
> 70 yr	1.59 (1.38–1.82)
Stage	
I	1.00 (Reference)
II	1.89 (1.55–2.29)
III–IV	3.71 (3.15–4.37)
RBT index	
Low	1.00 (Reference)
Intermediate	1.34 (1.11–1.61)
High	1.93 (1.62–2.31)
*AUC*	*0.76*

Since the CRP is not a blood parameter routinely collected in all centers, to improve the generalizability of our estimates, we re-calculated the RBT index without considering CRP and so classifying patients with low RBT index (score ≤ 9), intermediate (score between 10 and 14), and high (score ≥ 15). The results were consistent both overall, stratified by tumor stage, and among LM patients (Figure S3).

In the supplementary materials Table S6, we have described the multivariate Cox models for the RBT index excluding lymphocyte and neutrophil values step by step. In model 1, we used the RBT index as discussed above, complete with all three indices. In model 2, we modified the formula by excluding lymphocytes. In model 3, we excluded neutrophils, and in model 4, we excluded both values. Comparing these last three models to the original model containing lymphocytes, neutrophils, and their ratio, there is no difference in terms of model goodness, as reported by the likelihood ratio tests for non-nested Cox models.

## Discussion

The present study investigated the ability of inflammatory, immunologic and metabolic biomarkers, obtained from routine perioperative blood test, to predict survival of surgically resected LC patients. First, we observed that CRP and NLR (as surrogate markers of inflammatory status), monocytes, lymphocytes, and neutrophils (as markers of immunologic profile), hemoglobin, glycemia, and albumin levels (as markers of metabolic status) are independently associated with short-term (at 30- and 90 days) and long-term (at 5 years) survival. Then we combined these parameters to develop a composite prognostic score (RBT index) that was able to predict survival in patients undergoing surgical resection with curative intent for LC and LM. Finally, we showed that RBT index was still able to identify patients with good and poor prognosis after the exclusion of CRP, not always available in clinical practice as a routine examination. Using those simple humoral markers, we could stratify patients into three risk categories of potential value to target treatment decision-making.

In the era of precision medicine, there is an urgent need to develop easy tools to predict individual patients’ outcomes. The advent of molecular and genomic profiling provides reliable data for prognostication and tailoring the most appropriate targeted therapies. In particular, next-generation sequencing (NGS) has a pivotal role in the management of metastatic non-small cell lung cancer^25^. However, there are solid barriers to the widespread adoption of those tests, especially for resectable diseases^2,25^. The identification of reliable, fast, and cheap tools for assessing patients' prognosis is of paramount importance for LC, as well as for other solid tumors.

Metabolism and inflammation are closely related, especially in cancer patients^5,26,27^. Accumulating evidence supported the importance of metabolic status in predicting outcomes of patients with solid and haematological tumors^26,27^. Metabolic status impacted operative outcomes and the early postoperative period. Several studies highlighted that patients with poor performance status and poor metabolic reserves are more vulnerable to physical stressors, including surgery^27,28^. Albumin level is the most reliable and easy method of reflecting metabolic status^27,28^, and several studies corroborated our results supporting that albumin levels < 3 g/dl are associated with impaired outcomes^28,29^. Other authors evaluated different metabolic markers, including body composition measures such as the ratio between adipose (subcutaneous plus muscular fat) and lean tissues (muscular mass) assessed by CT scan^29,30^. Those data supported that the amount of lean and fat tissues influence preoperative morbidity and long-term survival^29,30^. Similarly, growing evidence suggested the association between inflammatory status and poor oncologic results^27–29^. Few studies tested CRP and IL-6 in predicting unfavorable results after surgical tumors resection^27–29^.

The aforementioned ALI and H-index provided compelling data useful for patients' prognostication^10–16^. Similarly, the Glasgow Prognostic Score (GPS) based on CRP and albumin levels might be a useful indicator of performance status and survival in cancer patients^17,31–33^. The GPS aims to measure the extent of aggressiveness of the tumor and the patient's prognosis, being an indicator of metabolic and inflammatory host response in cancer patients^31,32^. Our prognostic index has a similar endpoint to the GPS, but we aimed to provide more reliable prediction by the inclusion of other variables than albumin and CRP. CRP is an important hallmark of unfavorable outcomes in many diseases and in our prior experience we demonstrated that CRP levels predict 5-year survival in each and every LC stage^7,17,33^. However, only the combination of a high pre- and postoperative CRP predicted a significantly higher risk of 30- and 90-day LC mortality^7^. To overcome this restraint, we designed a simplified RBT index (without CRP) that maintained its predictive value, both in primary and metastatic settings (Figure S3).

Three points of the present study deserve to be further discussed. First, our validation cohort included patients with LMs represents an interesting clinical scenario. In fact, while the large spectrum of metastatic tumors analyzed here (Table S1) is characterized by very different biologic behaviors, the RBT index was able to predict short and long-term outcomes in the whole LM cohort. The presence of various types of solid tumors expands the potential use of RBT index to other cancer settings. Second, as aforementioned several blood scores have been proposed in the past with the purpose of patients’ prognostication. However, the clinical utility of these scores has been limited, and the long-term outcome prediction could not be utilized to tailor patient’s management. On the contrary, the prediction of a fourfold increase in perioperative mortality for patients with a high RBT index could be used to implement pre-habilitation and most robust postoperative supports^34^. Third, there is growing evidence that metabolic and inflammatory status have a significant impact on the outcome of patients with metastatic disease^35^. Several reports highlighted that cyclic fasting and calorie-restricted diets (with low-carbohydrate and low-protein intakes) in combination with systemic chemo and immunotherapy are associated with better antitumor activity in patients with advanced and metastatic cancers^35,36^. Our study highlighted that the metabolic and inflammatory profiles play an important role also in resectable LC, including earlier stages, as well as in oligometastatic diseases (LM). Hence, we might speculate that introducing changes in diet and exercise and/or offering anti-inflammatory drugs for tailored chemoprevention to the subset of high RBT index patients could provide a significant clinical benefit. Lung cancer patients with high RBT index represent an ideal target population for randomized clinical trial testing preoperative anti-inflammatory therapies to reduce 90-day mortality, as well as long-term multimodality approaches to improve life expectancy by reversing the unfavorable metabolic, immunologic and inflammatory status.

The inherent biases of the retrospective nature represent the main weaknesses of the present study. Other limitations include (1) the single-centre study design, (2) the lack of the evaluation of more specific histological features; and (3) the absence of data on the molecular/genomic profile of LCs and LMs. Our project aims to build a simple algorithm that might be useful to assess host-related prognostic determinants in patients undergoing thoracic surgery with curative intent. The main strength of this project is to evaluate a large cohort of LC patients and to validate our prognostic tool in a consecutive series of oligometastatic patients. Although our tool is not aiming to replace molecular and genomic profiling, such an inexpensive and easy-to-use digital repository of routine perioperative blood test might identify a compromised host profile to be combined with molecular patterns. The validation steps of our research, by testing RBT tool in patients with resectable oligometastatic diseases, confirmed its predictive value across different solid tumor types.

Our methodology could be applied at best to larger existing laboratory data bases, taking profit of machine learning technologies that require huge amounts of data^37–39^.

On the other hand, interventional studies targeting metabolic and inflammatory status, are needed to demonstrate that the short-term and long-term outcomes of high-risk patients can be improved by individually tailored intervention.

In conclusion, the present study showed that metabolic, immunologic and inflammatory biomarkers, assessed by simple routine perioperative blood examinations, can predict short- and long-term outcomes in patients with resectable LC and LM, and developed a simple and easily available tool for patients’ prognostication. The RBT index was able to predict 30- and 90-day mortality, as well as 5-year survival in different settings of thoracic oncology. In practical terms, RBT index represents a potentially useful tool to tailor pre- and postoperative management based on predicted individual risk, to reduce 90-day mortality in patients with resectable LC. Our external validation of RBT index by the heterogeneous LM cohort let us speculate that similar results might be achieved in other solid tumors, but a further external validation is warranted.

Hence, further larger prospective studies involving other tumor types, are needed to confirm the value of RBT index and test the feasibility of individual risk reduction by reversal of the unfavorable metabolic, immunologic and inflammatory status.

### Supplementary Information

Supplementary Information.

## Data Availability

Study data made available upon reasonable request to the corresponding author (UP).
