# Supplementary material for: Routine perioperative blood tests predict survival of resectable lung cancer
Source: Sci Rep. 2023 Oct 10;13:17072. doi: 10.1038/s41598-023-44308-y (PMC10564956; doi:10.1038/s41598-023-44308-y)
Supplement: Supplementary file 1 — Supplementary Information. [file 41598_2023_44308_MOESM1_ESM.docx]

**Supplementary material**

Routine perioperative blood tests predict survival of resectable lung cancer

This appendix has been provided by the authors to give readers additional information about their work.

TABLE OF CONTENTS

[Section 1: Detailed RBT index formula 2](#_Toc145506913)

[Section 2: Evaluation of the prognostic value of albumin three days after surgery vs albumin at baseline. 3](#_Toc145506914)

[Table S1: Primary tumor site in the lung metastasis cohort 4](#_Toc145506915)

[Table S2: 30-day and 90-day mortality 5](#_Toc145506916)

[Table S3. Socio-demographic, clinical, and inflammatory, immunological and metabolic markers description among survivors and the dead at five years. Patients with primary LC, N=2088. 6](#_Toc145506917)

[Table S4. Socio-demographic, and inflammatory, immunological and metabolic markers description among survivors and the dead at five years. Patients with lung metastases, N=1129. 7](#_Toc145506918)

[Table S5. Multivariate Cox proportional hazards model to assessed the prognostic value of age, sex, and RBT score in predicting 5-year mortality in the lung metastasis cohort, N=1129. 8](#_Toc145506919)

[Table S6. Test results for Multivariate Cox non-nested models 9](#_Toc145506920)

[Figure S1. The prognostic capacity of each blood parameter for OS at 5-year. 10](#_Toc145506921)

[Inflammatory markers: C-reactive Protein - neutrophil-lympocyte ratio 10](#_Toc145506922)

[Immunological markers: Lymphcytes - neutrophils - white blood cells - monocytes 11](#_Toc145506923)

[Metabolic markers: Albumine - glycemic index - hemoglobin - bilirubin 13](#_Toc145506924)

[Figure S2: ROC curves for RBT index in the Primary Lung cohort (A), and in the Lung metastases cohort (B). Panel C shows the Calibration plot for observed versus predicted overall survival at 5-years probabilities. 15](#_Toc145506925)

[Figure S3: Overall survival curves according to the modified RBT index cut-offs without CRP score; panel A: LC cohort; Panel B: LC cohort stratified for stage I disease; Panel C: LC cohort stratified for stage II-III disease; Panel D: LM cohort 16](#_Toc145506926)

# Section 1: Detailed RBT index formula

$$Routine Blood test \left( RBT \right)=Inflammatory score+Immunologic score+Metabolic score$$

$$Inflammatory score=CRP+NLR$$

$$Immunologic score=monocytes+lymphocytes+neutrophils$$

$$Metabolic score=haemoglobin+albumin+glycemia$$

$$CRP \geq3 mg/dL =5$$

$$NLR \geq2.9 =3$$

$$monocytes \geq0.32 x {10}^{3}/\mu L=4$$

$$lymphocytes<1.8 x {10}^{3}/\mu L=4$$

$$neutrophils \geq5 x {10}^{3}/\mu L=2$$

$haemoglobin<$*13.5 g/dL =5*

$$albumin<3.14 g/dL=5$$

$$glycemia \geq106 mg/dL=3$$

# Section 2: Evaluation of the prognostic value of albumin three days after surgery vs albumin at baseline.

Of 3217 patients, only 378 recorded albumin at baseline; all had albumin three days after surgery. The aim of this analysis was to assess if the prognostic value of albumin at baseline and three days after surgery in predicting the overall survival at 5-year, differed. We created a new variable as the difference between albumin 3-day after surgery and at baseline. The hazard ratio (HR) and the respective 95% confidence interval (95% CI) were estimated using Cox’s proportional hazard regression model. The results showed the same prognostic predicting value of albumin at 3-days after surgery and at baseline.

|  | HR | (95% CI) |
| --- | --- | --- |
| Difference | 0.94 | (0.77 - 1.16) |

# Table S1: Primary tumor site in the lung metastasis cohort

| **Primary tumor site** | **N** | **%** |
| --- | --- | --- |
| Sarcoma | 286 | 25.3 |
| Colon | 201 | 17.8 |
| Germ cell tumor | 107 | 9.5 |
| Rectum tumor | 94 | 8.3 |
| Melanoma | 86 | 7.6 |
| Kidney tumor | 64 | 5.7 |
| Breast cancer | 43 | 3.8 |
| HCC | 29 | 2.6 |
| Adenoid Cystic Carcinoma | 27 | 2.4 |
| Endocrine tumor | 20 | 1.8 |
| Other | 172 | 15.2 |
| Total | 1129 |  |

# Table S2: 30-day and 90-day mortality

|  | Patients | 30-day Mortality | p.value | 90-day  Mortality | p.value |
| --- | --- | --- | --- | --- | --- |
| Lung Cancer |  |  |  |  |  |
|  |  |  |  |  |  |
| Low RBT index | 743 | 0.5% | 0.0019^a^ | 1.20% | <.0001^b^ |
| Int. RBT index | 651 | 0.5% |  | 1.40% |  |
| High RBT index | 694 | 2.3% |  | 5.20% |  |
|  |  |  |  |  |  |
| Lung Metastases |  |  |  |  |  |
|  |  |  |  |  |  |
| Low RBT index | 527 | 0 | 0.1512^a^ | 0.40% | 0.0209^a^ |
| Int. RBT index | 245 | 0.40% |  | 0.40% |  |
| High RBT index | 357 | 0.60% |  | 2.20% |  |
|  |  |  |  |  |  |
| ^a^Fisher exact test |  |  |  |  |  |
| ^b^Chi-Squared test |  |  |  |  |  |

# Table S3. Socio-demographic, clinical, and inflammatory, immunological and metabolic markers description among survivors and the dead at five years. Patients with primary LC, N=2088.

|  |  | Total | | Survivors at 5 yr. | | Deaths at 5 yr. | | p-value* |
| --- | --- | --- | --- | --- | --- | --- | --- | --- |
|  |  | (N=2088) | | (N=1252) | | (N=836) | |  |
| Age at surgery in yr. - median (IQR) | | 67 | (13.1) | 66 | (12.6) | 69 | (13.4) | <.0001^a^ |
| Gender - n(%) |  |  |  |  |  |  |  |  |
|  | Female | 706 | (33.8) | 497 | (70.4) | 209 | (29.6) | <.0001^b^ |
|  | Male | 1382 | (66.2) | 755 | (54.6) | 627 | (45.4) |  |
| Stage - n(%) |  |  |  |  |  |  |  |  |
|  | I | 1002 | (48.0) | 771 | (79.6) | 231 | (23.1) | <.0001^b^ |
|  | II | 449 | (21.5) | 256 | (57.0) | 193 | (43.0) |  |
|  | III-IV | 637 | (30.5) | 225 | (35.3) | 412 | (64.7) |  |
| Inflammatory markers - n(%) |  |  |  |  |  |  |  |  |
|  | CRP ≥3 mg/dL | 1112 | (53.3) | 581 | (52.2) | 531 | (47.8) | <.0001^b^ |
|  | NLR ≥2.9 | 895 | (42.9) | 469 | (52.4) | 426 | (47.6) | <.0001^b^ |
| Immunology markers - n(%) |  |  |  |  |  |  |  |  |
|  | Mono. ≥0.32 x 10^3^/µL | 1381 | (66.1) | 778 | (56.3) | 603 | (43.7) | <.0001^b^ |
|  | Neu. ≥5 x 10^3^/µL | 836 | (40.0) | 455 | (54.4) | 381 | (45.6) | <.0001^b^ |
|  | Lymph.<1.8 x 10^3^/µL | 1127 | (54.0) | 631 | (56.0) | 496 | (44.0) | <.0001^b^ |
|  | WBC ≥8130 /µL | 671 | (32.1) | 371 | (55.3) | 300 | (44.7) | 0.0027^b^ |
| Metabolic markers - n(%) |  |  |  |  |  |  |  |  |
|  | Hb.<13.5 g/dL | 813 | (38.9) | 412 | (50.7) | 401 | (49.3) | <.0001^b^ |
|  | Alb.<3.14 g/dL | 784 | (37.5) | 389 | (49.6) | 395 | (50.4) | <.0001^b^ |
|  | Gly. ≥106 mg/dL | 741 | (35.5) | 409 | (55.2) | 332 | (44.8) | 0.0010^b^ |
|  | Bil.<0.5 mg/dL | 1027 | (49.2) | 593 | (57.7) | 434 | (42.3) | 0.0416^b^ |

*The p-value was calculated by comparison with the complementary class: females for gender, stage I, CRP <3 mg/dL, Lymph. ≥1.8 x 103/µL, Hb ≥13.5.

^a^Mann whitney U test

^b^Chi-square test

# Table S4. Socio-demographic, and inflammatory, immunological and metabolic markers description among survivors and the dead at five years. Patients with lung metastases, N=1129.

|  |  | Total | | Survivors at 5 yr. | | Deaths at 5 yr. | | p-value* |
| --- | --- | --- | --- | --- | --- | --- | --- | --- |
|  |  | (N=1129) | | (N=600) | | (N=529) | |  |
| Age at surgery in yr. - median (IQR) | | 58.1 | (20.8) | 57.6 | (23.3) | 58.5 | (19.7) | 0.0785^a^ |
| Gender - n(%) |  |  |  |  |  |  |  |  |
|  | Female | 466 | (41.3) | 247 | (53.0) | 219 | (47.0) | 0.9370^b^ |
|  | Male | 663 | (58.7) | 353 | (53.2) | 310 | (46.8) |  |
| Inflammatory markers - n(%) |  |  |  |  |  |  |  |  |
|  | CRP ≥3 mg/dL | 467 | (41.4) | 214 | (45.8) | 253 | (54.2) | <.0001^b^ |
|  | NLR ≥2.9 | 489 | (43.3) | 239 | (48.9) | 250 | (51.1) | 0.0120^b^ |
| Immunology markers - n(%) |  |  |  |  |  |  |  |  |
|  | Mono. ≥0.32 x 10^3^/µL | 565 | (50.0) | 295 | (52.2) | 270 | (47.8) | 0.5299^b^ |
|  | Neu. ≥5 x 10^3^/µL | 252 | (22.3) | 127 | (50.4) | 125 | (49.6) | 0.3213^b^ |
|  | Lymph.<1.8 x 10^3^/µL | 794 | (70.3) | 412 | (51.9) | 382 | (48.1) | 0.1932^b^ |
|  | WBC ≥8130 /µL | 170 | (15.1) | 88 | (51.8) | 82 | (48.2) | 0.6957^b^ |
| Metabolic markers - n(%) |  |  |  |  |  |  |  |  |
|  | Hb.<13.5 g/dL | 388 | (34.4) | 166 | (42.8) | 222 | (57.2) | <.0001^b^ |
|  | Alb.<3.14 g/dL | 188 | (16.7) | 80 | (42.6) | 108 | (57.4) | 0.0014^b^ |
|  | Gly. ≥106 mg/dL | 332 | (29.4) | 155 | (46.7) | 177 | (53.3) | 0.005^b^ |
|  | Bil.<0.5 mg/dL | 487 | (43.1) | 252 | (51.8) | 235 | (48.3) | 0.4120^b^ |

*The p-value was calculated by comparison with the complementary class: females for gender, CRP <3 mg/dL, Lymph. ≥1.8 x 103/µL, Hb ≥13.5.

^a^Mann whitney U test

^b^Chi-square test

# Table S5. Multivariate Cox proportional hazards model to assessed the prognostic value of age, sex, and RBT score in predicting 5-year mortality in the lung metastasis cohort, N=1129.

|  |  | HR (95% CI) |
| --- | --- | --- |
| Gender |  |  |
|  | Male | 1.00 (Reference) |
|  | Female | 0.97 (0.81-1.15) |
| Age |  |  |
|  | ≤70 yr. | 1.00 (Reference) |
|  | >70 yr. | 0.91 (0.74-1.13) |
| RBT index |  |  |
|  | Low | 1.00 (Reference) |
|  | Intermediate | 1.25 (0.99-1.58) |
|  | High | 1.88 (1.54-2.28) |

# Table S6. Test results for Multivariate Cox non-nested models

|  |  | MOD 1 | MOD 2 | MOD 3 | MOD 4 |
| --- | --- | --- | --- | --- | --- |
|  |  | aHR (95% CI) | aHR (95% CI) | aHR (95% CI) | aHR (95% CI) |
| Gender |  |  |  |  |  |
|  | Male | 1.00 (Reference) | 1.00 (Reference) | 1.00 (Reference) | 1.00 (Reference) |
|  | Female | 0.67 (0.57-0.78) | 0.68 (0.58-0.79) | 0.67 (0.57-0.79) | 0.68 (0.58-0.79) |
| Age |  |  |  |  |  |
|  | ≤70 yr. | 1.00 (Reference) | 1.00 (Reference) | 1.00 (Reference) | 1.00 (Reference) |
|  | >70 yr. | 1.59 (1.38-1.82) | 1.61 (1.41-1.85) | 1.56 (1.36-1.79) | 1.59 (1.39-1.83) |
| Stage |  |  |  |  |  |
|  | I | 1.00 (Reference) | 1.00 (Reference) | 1.00 (Reference) | 1.00 (Reference) |
|  | II | 1.89 (1.55-2.29) | 1.88 (1.55-2.28) | 1.91 (1.57-2.32) | 1.89 (1.56-2.30) |
|  | III-IV | 3.71 (3.15-4.37) | 3.75 (3.18-4.41) | 3.74 (3.18-4.41) | 3.74 (3.18-4.41) |
| RBT index |  |  |  |  |  |
|  | Low | 1.00 (Reference) | 1.00 (Reference) | 1.00 (Reference) | 1.00 (Reference) |
|  | Intermediate | 1.34 (1.11-1.61) | 1.30 (1.08-1.56) | 1.47 (1.23-1.77) | 1.31 (1.09-1.57) |
|  | High | 1.93 (1.62-2.31) | 2.06 (1.73-2.44) | 2.07 (1.72-2.49) | 1.95 (1.65-2.31) |
| *-2 LOG L* | | *11892.047* | *11875.117* | *11887.275* | *11884.433* |
| *Non-nested likelihood*  *ratio test* | | *Reference* | *MOD 1 vs MOD 2 pvalue=0.07671* | *MOD 1 vs MOD 3 pvalue=0.4333* | *MOD 1 vs MOD 4 pvalue=0.3073* |

MOD 1: RBT index

MOD 2: RBT index without lymphocytes

MOD 3: RBT index without neutrophils

MOD 4: RBT index without neutrophils and lymphocytes

# Figure S1. The prognostic capacity of each blood parameter for OS at 5-year.

## Inflammatory markers: C-reactive Protein - neutrophil-lympocyte ratio

## Immunological markers: Lymphcytes - neutrophils - white blood cells - monocytes

## Metabolic markers: Albumine - glycemic index - hemoglobin - bilirubin

# Figure S2: ROC curves for RBT index in the Primary Lung cohort (A), and in the Lung metastases cohort (B). Panel C shows the Calibration plot for observed versus predicted overall survival at 5-years probabilities.

# Figure S3: Overall survival curves according to the modified RBT index cut-offs without CRP score; panel A: LC cohort; Panel B: LC cohort stratified for stage I disease; Panel C: LC cohort stratified for stage II-IV disease; Panel D: LM cohort
